# IL‐1RA suppresses esophageal cancer cell growth by blocking IL‐1α

**DOI:** 10.1002/jcla.22903

**Published:** 2019-05-17

**Authors:** Sui Chen, Zhimin Shen, Zhun Liu, Lei Gao, Ziyang Han, Shaobin Yu, Mingqiang Kang

**Affiliations:** ^1^ Department of Thoracic Surgery Fujian Medical University Union Hospital Fuzhou China; ^2^ Key Laboratory of Ministry of Education for Gastrointestinal Cancer Fujian Medical University Fuzhou China; ^3^ Fujian Key Laboratory of Tumor Microbiology Fujian Medical University Fuzhou China

**Keywords:** esophageal cancer, IL‐1RA, VEGF

## Abstract

**Background:**

Interleukin‐1 promotes tumor angiogenesis through VEGF production. The interleukin‐1 receptor antagonist can suppress tumors by blocking this effect.

**Methods:**

Immunohistochemistry, WB, and gene sequencing were used to analyze the expression of IL‐1RA in esophageal cancer patients. WB was used to detect the expression of IL‐1RA and interleukin‐1α in esophageal cancer cells. Stable ESCC cell models overexpressing the IL‐1RA were constructed. Their cell functions were tested, and their effects on VEGF were examined.

**Results:**

IL‐1RA is downregulated in primary EC tumors, and this downregulation of IL‐1RA is closely related to TNM staging and survival prognosis. The overexpression of IL‐1RA increased the proliferation of KYSE410 EC cells, which have a high level of IL‐1α expression. Overexpression of IL‐1RA in KYSE410 cells promotes a decrease in the expression of VEGF‐A. However, IL‐1RA expression did not cause any changes in EC9706 cells with low IL‐1α expression.

**Conclusion:**

IL‐1RA acts as a tumor suppressor, and its deletion promotes tumor progression by increasing VEGF‐A expression in ESCC.

**Acronyms**
EC: esophageal cancer
ESCC: esophageal cancer cells
*IL‐1RA*: interleukin‐1 receptor antagonist
*IL‐1RN*: interleukin‐1 receptor antagonist gene
*IL‐1α*: interleukin‐1α
*VEGF*: vascular endothelial growth factor

## 1 INTRODUCTION

Esophageal cancer is the seventh most common form of malignant cancer worldwide and the sixth most common cause of cancer‐related death. Notable forms of esophageal cancer include adenocarcinoma of the esophagus and esophageal squamous cell carcinoma (ESCC).^1^, ^2^ Although current treatments, such as surgery and chemotherapy, have progressed esophageal cancer treatment, the 5‐year overall survival rate of ESCC is still low (20%‐30%) due to metastasis, recurrence, and resistance to chemoradiotherapy.^3^ Furthermore, the early symptoms and signs of esophageal cancer are difficult to detect; therefore, the disease is usually identified in the middle and late stages of pathogenesis. Subsequently, distant metastasis may occur, leading to poor prognosis.^4^, ^5^ Thus, exploring the mechanism of ESCC progression is critical to improve diagnosis, treatment, and prognosis.

Interleukin‐1 (IL‐1), a pro‐inflammatory chemokine, can promote the proliferation and differentiation of tumor cells via interactions with specific receptors on tumor cell membrane surfaces.^6^ IL‐1RA, IL‐1α, and IL‐1β are all members of the interleukin‐1 family. IL‐1α and IL‐1β bind to the same receptor and perform similar biological activities, such as promoting tumor growth and metastasis via enhanced angiogenesis. This activity is performed by the regulation of the expression of angiogenic factors, such as vascular endothelial growth factor (VEGF).^7^ IL‐1RA is a pan‐physiological inhibitor of IL‐1 that prevents the activation of IL‐1 receptors by inhibiting IL‐1 binding to its receptors. IL‐1 RA is a glycosylated protein of 23‐25 KDa. Cloned IL‐1RA DNA contains an open reading frame encoding 277 amino acids, including 25 N‐terminal amino acids present in the pro‐protein and the 152 amino acids present in the mature protein. The molecular weight is 17 115 Daltons, and the isoelectric point is pH 5.2. IL‐1RA has been connected with a variety of diseases, including cardiovascular disease, type II diabetes, carcinoma, and joint diseases.^8^, ^9^, ^10^ IL‐1RA can inhibit angiogenesis by blocking the activation of the IL‐1α/PI3K/NF‐κB pathway in IL‐1‐producing human colon cancer cells.^11^ Downregulation of IL‐1RA is correlated with the metastatic potential of gastric cancer through blockage of the IL‐1α/VEGF signaling pathways.^12^ IL‐1RN was confirmed to be downregulated in ESCC.^13^ However, the mechanism and function of IL‐1RA remains unclear.

## 2 MATERIALS AND METHODS

### 2.1 Clinical samples

#### 2.1.1 Acquisition of clinical samples and immunohistochemical analysis

Human esophageal cancer samples and homologous esophageal tissues were collected immediately after surgical resection at Union Hospital of Fujian Medical University (Fuzhou, China) from 2009 to 2011. All samples were immediately stored at −80°C for future paraffin embedding. Clinical staging was determined according to the American Joint Committee on Cancer (AJCC) eighth edition of EC TNM staging. Immunohistochemical staining with a rabbit anti‐IL‐1RA antibody (1:3200, Abcam, ab124692) was performed on formalin‐fixed, paraffin‐embedded tissue sections of 4‐5 µm, which were cut from tissue microarrays (TMAs) and placed on glass slides. A 5‐tiered scale was used to assess the degree of nuclear or cytoplasmic staining based on the average percentage of positive staining cells (0, ≤1% positive cells; 1, 1%‐10% positive cells; 2, 11%‐50% positive cells; 3, 51%‐80% positive cells; and 4, ≥80% positive cells), which was multiplied by the staining intensity (0, no staining; 1, weak staining, light yellow; 2, moderate staining, yellow‐brown; and 3, strong staining, brown) to obtain a score ranging from 0 to 12. A score <3.5 was considered as low IL‐1RA expression, while a score >3.5 was considered as high IL‐1RA expression. All of the tissues were analyzed by two pathologists.

#### 2.1.2 Western blot analysis

Tissues or cells were lysed in Western & IP cell lysis buffer (Beyotime) with PMSF (Amresco) for 30 minutes on ice at 4°C followed by centrifugation at 12 000 × *g* for 15 minutes at 4°C. The supernatants were collected, and the total protein concentration was measured using the BCA Protein Assay Kit (Thermo Scientific). Equimolar amounts of protein were loaded into each well and separated with 12% SDS‐PAGE. Then, proteins were transferred to a 0.45‐μm PVDF membrane (Amersham Hybond, GE Healthcare), which was blocked in 2% bovine serum albumin (Amresco) prior to overnight incubation overnight at 4°C with the following primary antibodies: rabbit anti‐IL‐1RA, rabbit anti‐IL‐1α (1:1000), mouse anti‐β‐actin (1:2000; Cell Signaling Technology), and VEGF‐A polyclonal antibody (1:1000, A41552). After three washes in TBST buffer lasting 10 minutes per wash, the membrane was incubated with secondary antibodies for 1 hour at room temperature. The blots were developed using enhanced chemiluminescence (Lulong Biotech).

#### **2.1.3** RNA extraction and real‐time quantitative PCR

2.1.3

Total RNA was extracted from cultured cells or frozen tissues using TRIzol reagent (Ambion), and 1 mg RNA was reverse transcribed using miScript Reverse Transcription Kit (Qiagen) for first complementary DNA strand synthesis. Quantitative PCR was performed using SYBR Premix EX Taq kit (Takara). Specific primers were used to detect the relative mRNA expression of IL‐1RA by the 2‐△△ Ct method. The expression level was normalized against endogenous GAPDH. All the primers were designed by BioSune Biotechnology Co., Ltd. (Shanghai).

### 2.2 Cell lines

Human EC cell lines KYSE410 and EC9706 were purchased from Hunan Fenghbio Biological Ltd, China. The cells were grown in RPMI‐DMEM (Gibco) medium supplemented with 10% FBS (Gibco) and incubated at 37°C in an atmosphere of 5% CO_2_.

#### 2.2.1 Plasmids and generation of stable ec cell lines

The opening reading frame of the human IL‐1RA gene was PCR‐amplified and cloned into the lentivirus expression vector pCDH‐CMV‐MCS‐EF1‐RFP‐Puro (System Biosciences). Either a recombinant plasmid or an empty vector was co‐transfected with packaging plasmids pMDL, pVSVG, and pRev into 293T cells. The supernatants were collected at 48 hours post‐transfection and used to infect KYSE410 and EC9706 cells cultured in 6‐cm dishes. Puromycin‐resistant clones were expanded into cell lines as IL‐1RA overexpressing cells (KYSE410‐PIL‐1RA or EC9706‐IL‐1RA) or empty control cells (KYSE410‐pCDH or EC9706‐pCDH). The protein expression levels of IL‐1RA were evaluated by Western blot analysis.

#### 2.2.2 Wound‐healing assay

Transfected cells were grown to 100% confluence in six‐well plates. The cell layers were scratched using a 20‐μL tip to form wound gaps, washed three times with phosphate‐buffered saline (PBS), and photographed at different time points. The cells were counted using a scale label to determine movement activity away from the original scratch location 48 hours after knockdown.

#### 2.2.3 Cell invasion assay

Cell invasion assays were performed using transwell membranes coated with Matrigel (NY, USA). Transfected cells were plated at a density of 5 × 10^5^ cells/well in the upper chamber with a serum‐free medium. FBS (10%) was added to the lower chamber as a chemoattractant. After 48 hours of incubation, the cells were stained with crystal violet for 5‐10 minutes. Finally, the invading cells were counted in five microscopic fields (200 × magnification).

#### 2.2.4 Colony formation assay

Stably transfected cells were harvested and seeded in six‐well plates at a density of 1 × 10^3^ cells/well. After 2 weeks, the cells were fixed in 3% methanol for 30 minutes and stained with 1% crystal violet for 10 minutes. The number of visible colonies was then counted using a phase contrast microscope.

#### 2.2.5 Cell proliferation assay

Cells in the logarithmic growth phase were planted in 96‐well plates at a density of 4 × 10^3^ cells/well. The next day, a Cell Counting Kit‐8 (CCK‐8; Donjido, Kumamoto, Japan) was used according to the manufacturer's instructions. The optical density (OD) was detected using a microplate reader (BioTek, VT, USA) at a fixed time each day for 6 days.

### 2.3 Statistical analysis

Statistical analysis was performed using SPSS 21.0 for Windows. All data used for the analysis were expressed as the means ± SDs from three independent experiments. The association between IL‐1RA expression and the clinicopathological parameters was analyzed using Pearson's chi‐square test and Spearman's rank‐order correlation. Survival curves were plotted using Kaplan‐Meier analysis. Differences were considered significant when *P*‐values were <0.05.

## 3 RESULTS

### 3.1 Downregulation of IL‐1RA in EC is correlated with poor prognosis

**Figure 1:**
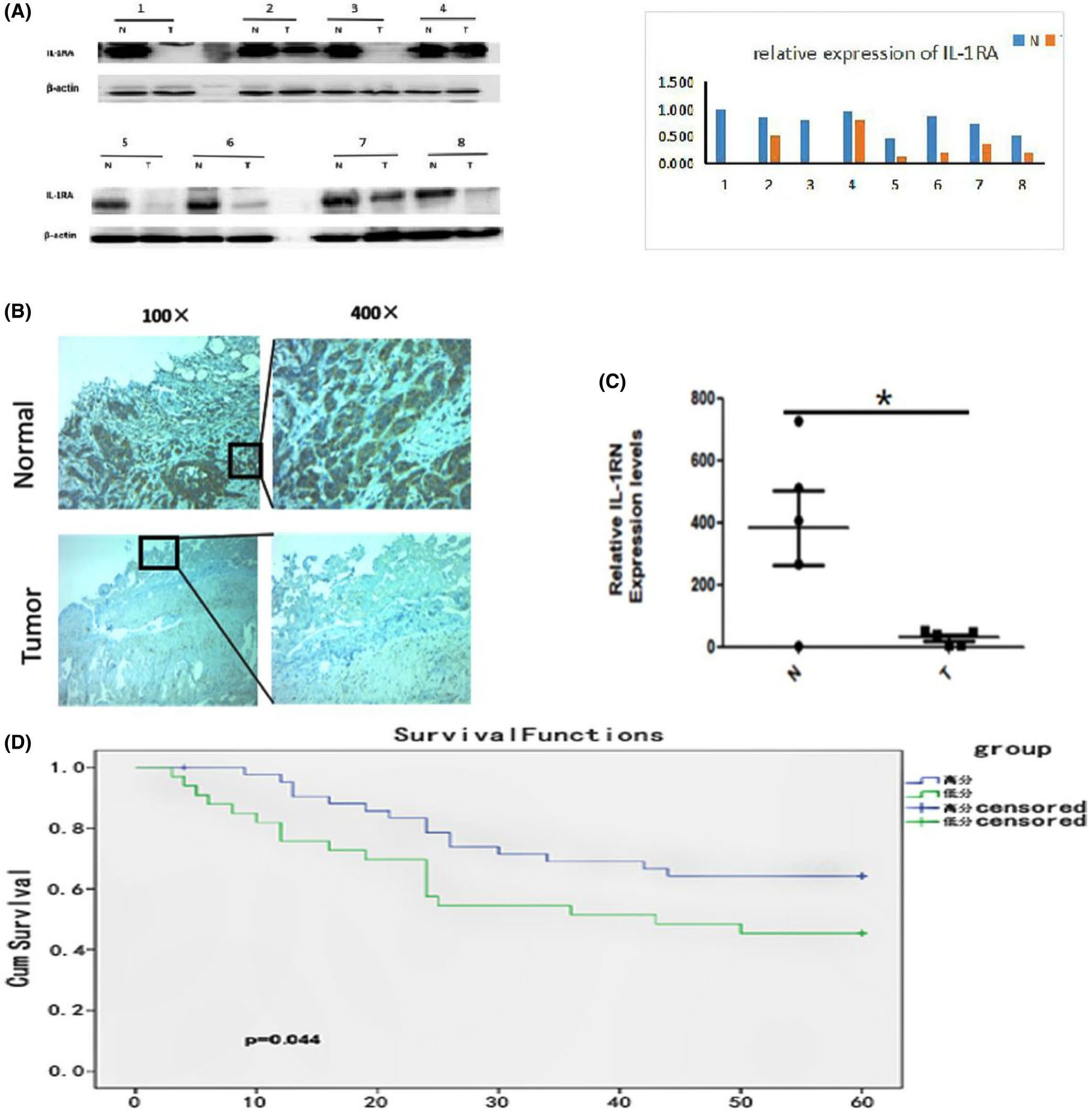
Clinical study: A, The expression of IL‐1RA was detected by Western blotting cancerous and paracancerous tissues from eight groups of patients. The results show that the expression of IL‐1RA in esophageal cancer tissues decreased compared to paracancerous tissue. B, Immunohistochemical staining of 76 tumor samples was performed to investigate the expression of IL‐1RA in paracancerous tissues and adjacent tissues and showed that the expression of IL‐1RA was significantly decreased in esophageal cancer patients (*P* = 0.000). C, The expression of the IL‐1RN gene in esophageal cancer and adjacent tissues was detected by RT‐PCR. The expression of IL‐1RN gene decreased in esophageal cancer tissues (*P* = 0.0031). D, Kaplan‐Meier survival analysis showing that downregulation of IL‐1RA in the 76 EC samples was associated with poorer overall survival (*P* = 0.044)

Seventy‐six tumor samples were analyzed by immunohistochemistry (IHC) to detect the prognostic value of IL‐1RA expression. As shown in representative Figure 1 and Table 1, IL‐1RA expression was significantly lower in the EC tumors than in the adjacent normal tissues. Western blot analysis and genetic testing also confirmed the expression of both IL‐1RA protein (Figure 1A) and mRNA (Figure 1B) decreased in the esophageal tumor tissues compared with the adjacent normal tissues. Pearson's chi‐square test evaluating IL‐1RA expression and clinicopathological features showed that the level of IL‐1RA expression in EC was associated with clinical staging; low expression of IL‐1RA meant advanced T staging, N staging, and TNM staging (*P* = 0.011; *P* = 0.049; *P* = 0.001, respectively) (Table 1). After confirming that IL‐1RA expression was downregulated in EC (*P* = 0.003), Kaplan‐Meier analysis was used to investigate the relationship between IL‐1RA expression assessed by IHC and patient outcome. As shown in Figure 1D, patients with low IL‐1RA‐expressing tumors had significantly shorter 5‐year survival than patients with high IL‐1RA‐expressing tumors (*P* = 0.044). Altogether, these results suggest that IL‐1RA may be a tumor suppressor and that a decrease in IL‐1RA expression can promote the progression of EC.

**Table 1:** Clinicopathological characteristics of 76 EC patients according to IL‐1RA

Item	IL‐1RA		*P* Value^a^
Normal vs Cancer	Low expression（n = 43）	High expression （n = 33）	
Normal	20	56	0.000^a^
Cancer	43	33	
Age			
<60	24	19	1.000
≥60	19	14	
Gender			
Females	8	7	0.780
Males	35	26	
TNM classification			
Ⅰ	11	1	0.001^a^
Ⅱ	19	8	
Ⅲ	13	24	
T classification			
T1	9	2	0.011^a^
T2	11	2	
T3	20	26	
T4	3	3	
N classification			
N0	19	8	0.049^a^
N1	8	15	
N2	4	16	
N3	2	4	
Tumor differentiation			
High	23	16	0.845
Middle	13	10	
Low	7	7	

a *P* value was determined using Pearson's chi‐square test.

### 3.2 IL‐1RA overexpression suppressed EC cell proliferation

**Figure 2:**
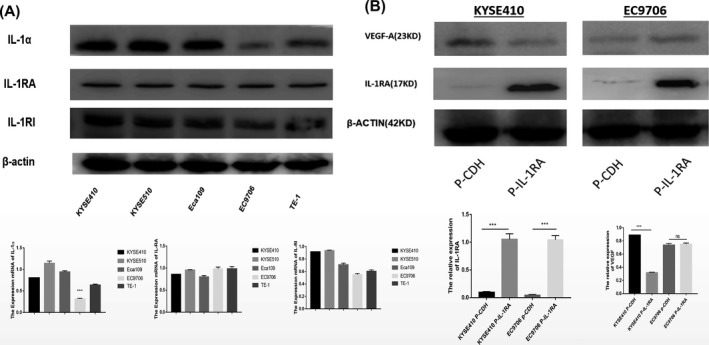
Cell study A, Western blotting (WB) detected the expression of IL‐1RA and IL‐1α in different esophageal squamous carcinoma cells. IL‐1RA is expressed in all esophageal squamous carcinoma cells, and IL‐1α is expressed at low levels in EC9706 cells. B, WB verified cells stably overexpressing IL‐1RA in esophageal squamous cell carcinoma. Changes in VEGF‐A expression were observed to correlate with the change in IL‐1RA expression. The results showed that for KYSE410 cells, the expression of VEGF‐A decreased with an increase in IL‐1RA expression, while for EC9706 cells, this result was not the case. In this case, the expression was not obvious

To investigate the biological functions of IL‐1RA in regulating ESCC, we first detected the expression of IL‐1RA and IL‐1α in EC cell lines (Figure 2A). Then, we used gene overexpression strategies to specifically increase the amount of IL‐1RA protein in the EC cell lines KYSE410 and EC9706. Stable overexpression of IL‐1RA in these cells was confirmed by Western blot analysis (Figure 2B). The overexpression of IL‐1RA did not produce any changes in the rate of migration of KYSE410 and EC9706 cells in vitro, as evaluated by wound‐healing assay (Figure 2C). Then, we compared the effect of IL‐1RA on cell proliferation using CCK‐8 and colony formation assays. As shown in Figure 2D and Figure 2E, proliferation was suppressed by overexpression of IL‐1RA in KYSE410 cells, which express IL‐1α. No difference was observed in EC9706 cells.

### 3.3 IL‐1RA suppressed ESCC proliferation via the IL‐1α/VEGF signaling pathway

**Figure 3:**
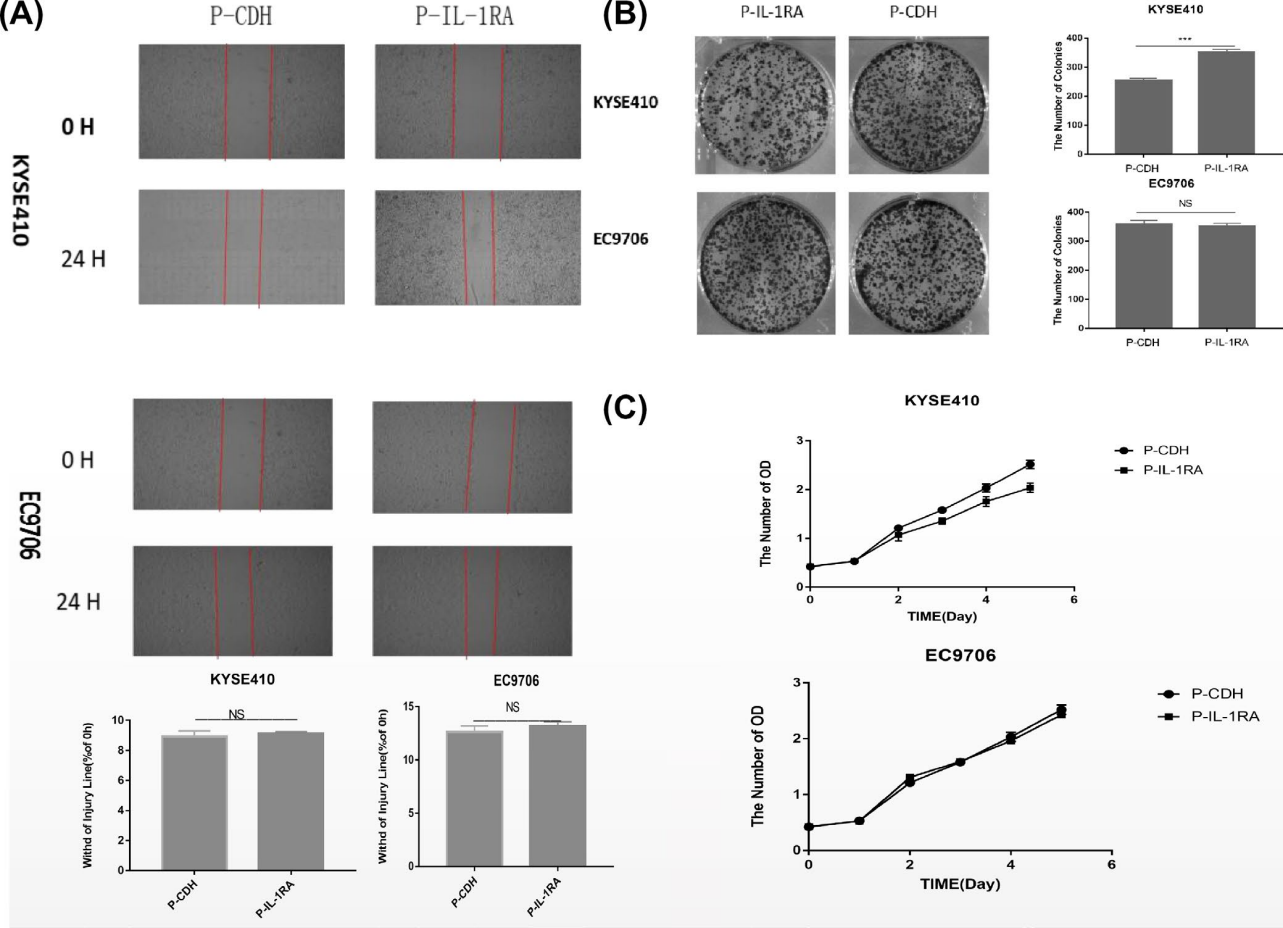
A, Cell scratch test to detect cell migration ability. IL‐1RA expression had no significant effect on cell migration ability. B, Cell plate clone assay to detect cell proliferation ability. IL‐1RA overexpression significantly reduced the proliferation of KYSE410 cells, but no effect was observed for the EC9706 cells. C, CCK‐8 assay to detect cell proliferation function. IL‐1RA overexpression significantly reduced the proliferation of KYSE410 cells (*P*＜0.05), but no effect was observed for EC9706 cells

To understand the molecular mechanisms underlying IL‐1RA‐induced inhibition of EC proliferation, we assessed the expression of VEGF‐A protein. VEGF‐A protein levels decreased in KYSE410 cells when IL‐1RA was overexpressed, but the VEGF‐A levels did not change in EC9706 cells, possibly due to the low expression of IL‐1α (Figure 2A, 2B). These data imply that IL‐1RA may suppress tumors by regulating the IL‐1α/VEGF signaling pathway in ESCC cells (Figure 3).

## 4 DISCUSSION

The results reported in this study provide evidence that the expression levels of IL‐1RA affect the behavior of EC. IL‐1RA is downregulated in primary EC tumors, and this downregulation of IL‐1RA is associated with advanced tumor stage, grade progression, and worse survival outcomes. In the cell models, the overexpression of IL‐1RA increased the proliferation of KYSE410 EC cells, which have high IL‐1α expression. Furthermore, the overexpression of IL‐1RA in the KYSE410 cells promotes a decrease in the expression of VEGF‐A. However, IL‐1RA does not increase the proliferation and expression of VEGF‐A in EC9706 cells with low IL‐1α expression. These results indicate that IL‐1RA acts as a tumor suppressor, and its deletion promotes tumor progression by increasing VEGF‐A expression.

The development of new blood vessels is a key pathway in the progression of solid tumors. Angiogenesis not only provides the tumor with oxygen and essential nutrients but also promotes the spread and proliferation of cancer cells.^14^ Angiogenesis is a complex, multi‐step process involving extracellular matrix remodeling, migration and proliferation of endothelial cells, and capillary formation.^15^ VEGF is the prototypical angiogenic stimulating molecule that has been implicated in several steps of the angiogenic process. Increasing VEGF expression is directly proportional to increasing tumor growth and metastasis by promoting angiogenesis and increasing vascular permeability.^16^ The role of VEGF in angiogenesis was further confirmed by the effects of hypoxia and several indirect effects of pro‐angiogenic factors, which were able to further increase the synthesis of VEGF.^17^, ^18^ It is important that VEGF has also been associated with tumor progression by stimulating angiogenesis in human esophageal carcinoma.^19^, ^20^

IL‐1RA is a member of the interleukin‐1 family. In contrast to IL‐1α and IL‐1β, which promote tumor progression through inflammation, IL‐1RA possesses a tumor‐suppressing effect by blocking the binding of IL‐1 to its target receptor [11,12]. Increasing amounts of evidence have shown that the microenvironment of tumors, including inflammation, plays an important role in the development and progression of cancer. Furthermore, inflammatory cytokine–mediated signaling pathways are involved in the malignant transformation of tumor cells.^21^, ^22^ In addition, IL‐1 produced by tumor‐associated macrophages (TAMs) is an inflammatory cytokine and can lead to the expression of NF‐κB‐regulated genes, such as VEGF‐A, in chondrosarcoma cells.^23^, ^24^ Our study showed that KYSE410 cells have a high expression of IL‐1α; when IL‐1RA was overexpressed, the expression of VEGF‐A, which has been reported to promote angiogenesis, was reduced. The results of CCK‐8 and colony formation assays showed that IL‐1RA inhibits the proliferation of KYSE410 cells. Finally, no difference in VEGF‐A expression or cell proliferation was observed upon overexpression of IL‐1RA in the EC9706 cells, which naturally have low IL‐1α expression. All our experiments indicate that IL‐1RA may inhibit the angiogenesis of EC cells by inhibiting the IL‐1α/VEGF signaling pathway. In addition, anakinra (Kineret), an IL‐1 receptor antagonist that blocks the biologic activity of IL‐1, has been confirmed to be safe to inject into the human body.^25^, ^26^ Thus, anakinra has been approved as a clinical drug for the treatment of symptoms of rheumatoid arthritis and hidradenitis suppurative.^27^, ^28^ In summary, our study found that IL‐1RA acted as a tumor suppressor in EC via regulation of the IL‐1α/VEGF signaling pathway; therefore, IL‐1RA might serve as a potential molecular target for EC treatments.
